# Unintended consequences of an ‘all-clear’ diagnosis for potential cancer symptoms: a nested qualitative interview study with primary care patients

**DOI:** 10.3399/bjgp16X683845

**Published:** 2016-02-08

**Authors:** Cristina Renzi, Katriina L Whitaker, Kelly Winstanley, Susanne Cromme, Jane Wardle

**Affiliations:** Health Behaviour Research Centre, University College London, London.; School of Health Sciences, University of Surrey, Guildford.; Health Behaviour Research Centre, University College London, London.; Health Behaviour Research Centre, University College London, London.; Health Behaviour Research Centre, University College London, London.

**Keywords:** cancer, delay, diagnostic investigations, diagnosis, help seeking, symptoms

## Abstract

**Background:**

Nine out of 10 patients undergoing urgent cancer investigations receive an ‘all-clear’ diagnosis.

**Aim:**

A qualitative approach was used to evaluate the impact of investigations that did not result in cancer diagnosis on subsequent symptom attribution and help seeking for recurrent or new possible cancer symptoms.

**Design and setting:**

A survey of symptoms, help seeking, and past investigations was sent to 4913 individuals aged ≥50 years from four UK general practices. Of 2042 responders, 62 participants were recruited still reporting at least one cancer ‘alarm’ symptom in a 3-month follow-up survey for a nested in-depth interview study (ensuring variation in sociodemographic characteristics).

**Method:**

Framework analysis was used to examine the in-depth semi-structured interviews and identify themes related to previous health investigations.

**Results:**

Interviewees were on average 65 years old, and 90% reported investigations within the previous 2 years. Most often they reported gastrointestinal, urinary, and respiratory symptoms, and 42% had waited ≥3 months before help seeking. Reassurance from a previous non-cancer diagnosis explained delays in help seeking even if symptoms persisted or new symptoms developed months or years later. Others were worried about appearing hypochondriacal or that they would not be taken seriously if they returned to the doctor.

**Conclusion:**

An all-clear diagnosis can influence help seeking for months or even years in case of new or recurrent alarm symptoms. Considering the increasing number of people undergoing investigations and receiving an all-clear, it is paramount to limit unintended consequences by providing appropriate information and support. Specific issues are identified that could be addressed.

## INTRODUCTION

Delays in seeking medical attention for possible cancer symptoms, as well as delays occurring along the diagnostic pathway, contribute to late-stage cancer diagnosis and poorer survival.^1^–^4^ To improve early diagnosis, numerous initiatives encourage symptomatic presentation and prompt diagnostic investigations.^5^–^9^ Given that nine out of 10 symptomatic patients undergoing urgent cancer investigations receive an all-clear or non-cancer diagnosis,^10^ possible unintended consequences that could affect large numbers of people should not be neglected. Previous studies have shown that an all-clear or a benign diagnosis after investigations (also called a ‘false alarm’) can have negative psychological effects.^11^–^14^ There is also some evidence that it can be associated with delayed cancer diagnosis in case of subsequent cancer symptoms.^3^,^4^,^15^–^20^ The processes linking an all-clear diagnosis to subsequent delays have only been marginally explored, however. Limited evidence is available on the cognitive and emotional factors involved in symptom attribution and help seeking after an all-clear diagnosis. A recent systematic review^21^ provided some information, but it was limited by mainly relying on small retrospective studies of patients with cancer diagnosed after symptomatic presentation; most studies do not specifically aim to examine the impact of an all-clear diagnosis on help seeking. Collecting information after a patient has received a cancer diagnosis can be susceptible to recall bias,^22^ as well as being restricted to people who, following the diagnosis, are well enough to be interviewed. Despite these limitations, the available data suggest that over-reassurance or under-support after an all-clear can influence delays in help seeking for cancer symptoms.

Some studies on false alarms in the screening context, rather than in symptomatic individuals, have also reported delayed help seeking among females who later developed interval breast cancer.^22^ Findings referring to screened or symptomatic individuals, however, are not directly transferable.^23^ Thus, this study will focus exclusively on an all-clear diagnosis following a symptomatic presentation.

The importance of remaining vigilant after an all-clear and of seeking help promptly for future potential cancer symptoms is highlighted; the risk of cancer during the years following an all-clear diagnosis is not insignificant.^24^–^27^ For example, up to 8% of colorectal cancers are diagnosed within 3–5 years of a negative colonoscopy,^28^ and, based on a UK study, the diagnostic yield of a second urgent referral, despite being lower than the first referral, is still noteworthy (5% compared with 10%).^24^ Similar considerations apply to other cancers, with the risks varying depending on the type and timing of the diagnostic tests previously performed.^27^,^29^

**How this fits in**

Nine out of ten patients undergoing urgent investigations for cancer receive an ‘all-clear’ or ‘non-cancer’ diagnosis. There is limited evidence on the unintended consequences of an ‘all-clear’ diagnosis in case patients later develop new or recurrent ‘alarm’ symptoms. This study highlights that an ‘all-clear’ diagnosis can lead to delays in help seeking for new or recurrent ‘alarm’ symptoms. Specific issues are identified that could be addressed for limiting delays after an all-clear diagnosis.

According to the model of pathways to treatment,^30^ the process to diagnosis is dynamic and includes ‘forward and backward movements’, with both patients and healthcare providers needing to reappraise symptoms repeatedly over time. Factors influencing the progress through the diagnostic pathways include patient-, health care-, and disease-related factors. Following an all-clear diagnosis, emotional and cognitive factors, as well as the circumstances surrounding the investigations, could play a role in influencing subsequent symptom attribution and help seeking. In line with the social cognitive theory (SCT),^31^,^32^ the decision to seek help can be influenced by a patient’s perceived ability to discuss a symptom and receive help (‘self-efficacy’), and sociostructural barriers and opportunities, as well as ‘outcome expectations’ (for example, if a patient expects that seeing a doctor will improve prognosis or control symptoms). Self-efficacy is affected by previous experiences, in addition to social models and social persuasion. Experiences with health investigations can, therefore, be expected to play an important role in influencing subsequent help seeking for cancer symptoms.

The objective of the present study was to evaluate the impact of a previous health investigation that excluded cancer on symptom attribution and help seeking among individuals with new or recurrent possible cancer symptoms. With the aim of overcoming the limitations of previous studies, the effect of an all-clear diagnosis was examined using a qualitative interview study nested within a large primary care survey of adults currently experiencing potential cancer ‘alarm’ symptoms persisting for at least 3 months.

## METHOD

A survey of symptoms, help seeking, and past healthcare experiences was sent to 4913 individuals aged ≥50 years from four general practices in England. A total of 2042 individuals responded to the survey; responders included slightly more males and older age groups (54% male; mean age 65 years) compared with non-responders (49% male; mean age 63 years). From the 2042 responders, those reporting at least one of 14 cancer ‘alarm’ symptoms were identified (*n* = 936), and they were sent a follow-up questionnaire after 3 months. Participants who were still reporting the ‘alarm’ symptom in the follow-up questionnaire (*n* = 271), and consented to contact (*n* = 215), were eligible to take part in a nested qualitative interview study. The first 144 responders meeting the study criteria were invited to participate in in-depth semi-structured interviews, with a response rate of 60% (*n* = 86/144). Invitees for the qualitative study were purposively selected based on sex, age, and geographic area of residence. After completing 62 in-depth interviews, data saturation and satisfactory variation in sociodemographic characteristics were achieved and further interviews were suspended.

Exclusion criteria were a previous cancer diagnosis and severe physical or mental health problems according to GP records. The symptom list was developed based on the Cancer Awareness Measure (CAM) and the *Be Clear on Cancer* campaigns as previously described.^33^ Details of the study methods are described elsewhere.^33^ Three investigators performed the interviews in participants’ homes (*n* = 8), UCL offices (*n* = 15), or over the telephone (*n* = 39), as participants preferred. The interviews lasted on average 42 minutes (range 22–66 minutes). Initially responders were asked to describe in their own words symptoms experienced over the previous 3 months, describing the course of symptoms, their thoughts, help-seeking behaviour, and experiences with health care. An interview guide was used to explore topics that were not mentioned spontaneously (Appendix 1). During the final part of the interview, prompts were used to elicit more in-depth descriptions regarding specific topics such as experiences with health investigations and help seeking after an all-clear diagnosis. For example:

‘*Some people having had investigations in the past wait before seeing a doctor again if symptoms continue or if they have new symptoms. Have you experienced something similar?*’

These questions helped to elicit the recall of actual experiences of study participants. Actual experiences of all-clear diagnoses and subsequent help-seeking behaviour reported by participants were the subject of the present study. The interviewers did not use the word ‘cancer’ unless the participant mentioned it during the interview; symptoms and help seeking were discussed in a broader health context. This aimed to examine symptom interpretation in everyday life,^34^ as people with ‘alarm’ symptoms rarely attribute them to cancer.^35^ If symptoms and help seeking are discussed in the context of cancer, people may be more likely to report minimal help-seeking delay, as suggested by studies focusing on other important diseases. For example, there is evidence that mentioning the word ‘stroke’ was associated with shorter anticipated delay than when stroke symptoms were discussed using only descriptive information.^36^ The researchers’ backgrounds in psychology or public health allowed them to discuss symptoms and help seeking in a broader context.

Interviews were audiotaped, transcribed verbatim, and anonymised following standard procedures.^37^ Transcriptions were compared with sections of each recording by one investigator and deemed accurate. Framework analysis was used to examine experiences of an all-clear diagnosis (defined here as investigations that resulted in a benign or any non-cancer diagnosis) and the impact on subsequent symptom attribution and help seeking. The initial ‘familiarisation’ with the data was achieved by repeatedly reading the transcripts. An index of major themes and subthemes was created by analysing and comparing identified themes within and across interviews in an iterative process. The data were arranged within a thematic framework to explore the relationships between the identified concepts.^38^ The framework and theme classification was further refined, drawing on the findings of a recent systematic review on the subject matter.^21^ Data extraction was performed by three investigators, and discussions between all coauthors allowed identification of key concepts and condensing of the number of themes and subthemes. Any disagreement was resolved through discussions and re-evaluation of the transcripts. NVivo 9.0 software was used to annotate the transcripts before mapping and interpretation of the data. Stata 13 software was used to analyse sociodemographic and clinical characteristics.

Participants’ accounts of symptom attribution and help seeking reported during the qualitative interviews were examined according to previous experiences of an all-clear diagnosis (considering also the time since the all-clear and the type of diagnostic investigations), as well as symptom characteristics. For the purpose of this study, where participants had multiple symptoms, only the symptom(s) mentioned in relation to a previous all-clear were examined in-depth.

Quotes from the interviews are used to illustrate the findings. To contextualise the interview extracts, details on participant’s age, sex, most relevant symptom, and type and estimated time since investigations are provided. The aim was not to evaluate the exact time intervals between an all-clear diagnosis, new symptom onset, and subsequent help seeking, but rather to gain an understanding of the process linking a previous all-clear to symptom attribution and help seeking for current symptoms. Thus, time intervals before help seeking are reported for illustrative purposes only, and to provide a richer representation of the findings. For the purpose of this study, the term ‘delayed’ help seeking refers to situations when participants reported waiting for several weeks or months before seeking medical advice despite noticing symptoms. It does not refer to a time interval with a pre-specified duration, as it was beyond the scope of this study to impose any cut-off points for excessively long intervals, particularly considering the variety of symptoms and diagnostic investigations.

## RESULTS

The characteristics of the 62 study participants are shown in Table 1. Interviewees were on average 65 years old, 53% were male, and 45% had university-level education. Sociodemographic characteristics were in line with the overall survey sample (*n* = 2042).^33^ A range of symptoms (persisting for at least 3 months) were reported, most commonly respiratory, gastrointestinal (GI), and urinary symptoms, and 42% of responders had waited ≥3 months before help seeking. Of the sample, 90% (*n* = 56) reported a diagnostic test resulting in an all-clear diagnosis within the previous 2 years, most often ultrasound scan (44%), CT/MRI (34%), and chest X-ray (28%). The subsequent findings refer to these patients having experienced an investigation with an all-clear diagnosis.

**Table 1.** Characteristics of study participants (*N* = 62)

**Characteristics**		***N* (%)**
**Age, years, mean (range)**		65 (50.3–92.7)

**Sex**	Male	33 (53.2)
	Female	29 (46.8)

**Ethnic group**	White British	57 (91.9)
	Other white background	3 (4.8)
	Other	2 (3.2)

**Education level^a^**	University degree	28 (45.2)
	Higher qualification below degree	13 (21)
	Lower qualification	17 (27.4)
	Other	4 (6.5)

**Most common symptoms in last 3 months^b^**	Persistent cough or hoarseness	17 (27.4)
	Abdominal bloating	16 (25.8)
	Persistent change in bladder habit	15 (24.2)
	Persistent change in bowel habits	12 (19.4)
	Unexplained pain	12 (19.4)
	Change in the appearance of a mole	7 (11.3)
	Rectal bleeding	4 (6.5)
	Unexplained lump	4 (6.5)

**Diagnostic investigation during the previous 2 years**	During the previous 3 months	25 (40.3)
	3–6 months ago	10 (16.1)
	6–12 months ago	10 (16.1)
	12–24 months ago	4 (6.5)
	Date unknown	7 (11.3)
	No investigation	6 (9.7)

a Lower qualification = O Level/GCSE, no formal qualifications.

b More than one symptom could be reported.

Even though the interviewers did not use the word cancer, to avoid influencing responses, all participants spontaneously referred to cancer at some point during the interview. Cancer was mentioned as something they had thought about either in the past or at present.

Despite variations in symptoms and sociodemographic characteristics of interviewees, common themes emerged in relation to an all-clear diagnosis. A previous all-clear was a common explanation for attributing current potential cancer symptoms to a benign diagnosis and delaying help seeking. In a more limited number of cases, a previous all-clear was a factor encouraging prompt help seeking for their current symptom.

Two overarching themes emerged in the interviews as influences on delay: over-reassurance and under-support. For each of these two themes, a number of subthemes were identified, as summarised in Box 1 (Appendix 2).

Box 1. **Factors influencing delayed help seeking in relation to a previous all-clear diagnosis**

**Table:** 

**Themes**	**Subthemes**
Over-reassurance	Symptom normalised or attributed to a previous benign diagnosis
	Perception of understanding the problem based on previous experience
	Trust in diagnostic tests overruled bodily sensations
Under-support	Worry about appearing hypochondriacal/making a fuss
	Not wanting to waste doctor’s time/bother the doctor
	Humiliation, not being taken seriously
	Symptoms previously dismissed
	Frustration, resignation, doctor unable to help
	Perception of GP as gatekeeper
	Lack of communication/information
	Anxiety, fear, embarrassment

### Over-reassurance

Reassurance from a previous non-cancer diagnosis was one of the most relevant themes explaining delays in help seeking for current symptoms. Having been reassured by a previous health investigation, some patients normalised their recurrent/persistent symptoms:

‘Well, it reassured me that it wasn’t any malignancy … I mean, they are not so bad that I can’t live with them; I just accept that that’s the way they are …’

(F, 65 years; persistent GI symptoms for which she has not sought medical advice; colonoscopy 7 years earlier)

Even in the case of new signs/symptoms affecting the same organ or site, some patients reported that they did not need professional advice, as they had the perception to understand the problem:

‘If you think you understand, oh I’ve had this before, you will put it off … I mean, it’s like those moles, after the first one arrived, the second one … I thought that’s going to go away, I’ve seen this before …’

(M, 54 years; skin lesion for which he delayed help seeking)

Some participants had an ‘instinct’ that the symptom may be ‘linked to cancer’ but trust in earlier tests made them disregard their bodily sensations:

‘… when I say I’m always worried it could be linked to cancer or some other awful disease, I always try to not pay attention to myself because I feel as if I could be just being overdramatic … So I don’t trust my own instincts in that regard, I think I’m just overreacting … Yes, I think that’s accepting the word of the doctor or the tests … I would just assume it was the same …’

(F, 50 years; recurrent GI symptoms for which she has not sought medical advice after a diagnosis of viral GI infection 12 months earlier)

Another participant reported:

‘ … if there’s nothing there on the X-ray then, perhaps, it’s all in your head … So I think having a clear X-ray always puts your mind at rest, and you would be silly not to take that into account …’

(F, 65 years; worsening respiratory symptoms, for which she did not seek help during the last 2 years; previously diagnosed with COPD)

### Under-support

Having received an all-clear, some people worried they would waste the doctor’s time or they would be considered hypochondriacal if they returned to the doctor:

‘ … I had, in the back of my mind, if I went to him and said, “Look, I’ve got that cough back again”, I suppose … he would have said, “Well, we’ve done all the tests, and everything is clear.”’

(M, 55 years; persistent cough for which he delayed help seeking after a previous chest X-ray)

The symptoms would have to interfere substantially with a patient’s life to overcome the worry of appearing hypochondriacal:

*‘… they always examine you like you’re being* [a] *hypochondriac … so to go a second time the symptom would have to be real and something that I would have to deal with.’*

(M, 68 years; weight loss, for which he delayed help seeking after previous all-clear)

Negative healthcare experiences in themselves were also a reason for delay, in some cases even years later:

‘ … from the GP to whatever, wasn’t, on the whole, very good … Well, the consultant, particularly in the hospital, wasn’t at all good. It made me, for a long time, feel I never wanted to go in the hospital again.’

(F, 71 years; recurrent GI symptoms for which she delayed help seeking)

Some reported that their symptoms were dismissed or they felt humiliated, which prevented them from returning to their doctor despite persistent symptoms.

‘And so I don’t go back because I feel like that … like, for one thing that I went for, she just laughed at me.’

(F, 62 years; recurrent pain, history of breast lump with all-clear after mammogram; delayed help seeking)

Participants often reported a sense of resignation and loss of trust in the healthcare providers as they felt that they were considered as somebody making a fuss:

‘ … I lost faith in them and didn’t go back … well, I do feel as though I am … not annoying them, but “oh not you again”, sort of thing … Well, when you go so many times …’

(M, 62 years; recurrent abdominal pain and bloating for which he delayed help seeking for 12 months after all-clear)

Some patients had the perception that the GP’s role as a ‘local’ doctor who, knowing them, would be able to consider the evolution of their health problems over time, had been essentially replaced by a gatekeeper role:

‘No, I thought if two specialists have had a look … it’s difficult to get over to them how much of an aggravation it actually is … there’s no attempt to interrelate or look back at anything, they have a fixed idea …’

(M, 69 years; recurrent skin problem for which he delayed help seeking after previous benign diagnosis)

This, combined with the perception that GPs have insufficient specialist knowledge, made some people feel that it would not be helpful to return to the GP:

‘I think it was taken seriously, but I don’t think they were competent to diagnose it … I just think of them as gatekeepers … I do think if one was able, when you have continuing conditions, if you are able to just go back to the specialist rather than have to go through your GP every time …’

(F, 54 years; respiratory problems, delayed help seeking for recurrent symptoms)

A further relevant theme was a lack of communication/information, leaving patients unsure and confused about what to do next. They felt that they were not given the opportunity to discuss their concerns at the time of the investigation, which left them worrying about their symptoms and the results of the investigations:

‘I think … after the scan I’d been given 5 minutes to think about it … but it was like, while I’m still lying on the table, she says, “We haven’t found anything.” … Because of the whole way it happened, I am reluctant to go back to the doctor again.’

(M, 58 years; persistent testicular lump for which he delayed help seeking for 3 months after an ultrasound with an all-clear diagnosis, despite worrying about test results)

Another participant said:

‘… if there was more time, if they could explain why they are giving you this … But I really go not to have something cured if it doesn’t need it … but to have advice on what it is.’

(F, 65 years; recurrent difficulty swallowing and lesions on scalp; delayed help seeking for years after all-clear)

Previous false alarms sometimes appeared to have been a missed opportunity for providing patients with advice on the importance of paying attention to bodily changes and encouraging future help seeking. For example, delayed help seeking for vaginal bleeding was reported by a female treated for vaginal polyps in the past, but who had not received explanations at that time:

Interviewer: ‘Did you feel that you understood your symptom better following that experience?’

Participant: ‘No. It was just … what do you say? If you have toothache, you go and get your tooth out. It’s a bit like that.’ (F, 63 years; vaginal bleeding for which she did not seek help for the last 6 months; vaginal polyp removed 4 years earlier)

In addition to over-reassurance and under-support from the healthcare provider, other patient factors influenced help seeking in conjunction with an all-clear diagnosis. These included older age, a fatalistic attitude, comorbidities, or specific symptom characteristics. Fear of diagnostic investigations or embarrassment in the case of investigations involving intimate body parts were rarely mentioned as deterrents.

### An all-clear diagnosis as a motivator for help seeking

In a small number of cases, previous non-cancer diagnoses that prevented more serious problems encouraged patients to seek help promptly:

‘… before I had that hemicolectomy, she said if I’d left it any longer it could have ruptured and that would have been worse … So if I’m worried, I go to the doctor’s.’

(F, 62 years; recurrent GI symptoms, for which she sought help promptly; operated on colon 5 years earlier for suspected cancer but received a benign diagnosis)

A trusting and friendly relationship with the doctor also encouraged patients to return:

‘I’d go to my GP, and say, “What do you think it is?” And he would, knowing me as he does, say, “Go and have an X-ray”, or something like that … I believe if it’s there and you’ve got discomfort, however minor it can be … you should go … He’s established an act of confidence with me.’

(M, 83 years; recurrent pain; has had repeated investigations and GP visits; no delay)

Participants who received specific advice on symptom monitoring felt they had their health under control:

‘… where there were suspect moles. They weren’t malignant, but they were capable of going malignant … what they did, they took photographs of my body. And every 6 months I’m supposed to look at it … I look at myself and make sure, you know, if I’ve lumps and bumps …’

(M, 67 years; had previous moles removed and is monitoring other moles as recommended; no delay)

## DISCUSSION

### Summary

The findings of this study suggest that an all-clear diagnosis can have unintended consequences influencing subsequent symptom attribution and help seeking. Reassurance and a false sense of security from a non-cancer diagnosis were common explanations for delaying help seeking for alarm symptoms that developed months or even years later. Trust in diagnostic tests or ‘the word of the doctor’ appeared to have an important effect on normalising symptoms and some people thought they should disregard their bodily sensations despite persisting or worsening symptoms. Many participants worried about appearing hypochondriacal or that they would not be taken seriously, especially if symptoms were vague or did not significantly interfere with daily life. The perception of insufficient support from healthcare providers, including inadequate time for doctor– patient communication and lack of advice on what to do in case of recurrent symptoms, prevented people returning to the doctor. Previous investigations were sometimes a missed opportunity for improving patients’ awareness on the importance of prompt help seeking and for supporting them in case of future cancer alarm symptoms.

### Strengths and limitations

Compared with previous research, the present study has provided a more comprehensive and detailed picture of the complex relationships between diagnostic investigations, symptom awareness, and help seeking in case of new or recurrent cancer alarm symptoms. This has been possible thanks to the in-depth qualitative interviews specifically designed to investigate the subject matter, and thanks to the inclusion of a relatively large sample of symptomatic patients not (yet) diagnosed with cancer. In particular, specific issues have been identified that could be addressed to limit possible unintended consequences after an all-clear diagnosis. By interviewing people currently experiencing symptoms but not diagnosed with cancer and avoiding reference to cancer during the interview, unless mentioned by participants, the risk of post-hoc rationalisation and recall bias was limited as far as possible.

Inaccuracy of patients’ recall of help seeking and reasons for delay cannot be entirely excluded and could limit the study findings. Even though study participants were selected from a wider population survey, selection bias may have influenced the findings, as in-depth interviews could only be performed with individuals who responded to the wider survey and who agreed to be interviewed. However, the sociodemographic characteristics of interviewees were sufficiently broad and they were in line with the survey sample.

The qualitative study did not allow estimation of the strength of the association between specific factors and delayed help seeking, and future large prospective studies are needed. In this study, people with a variety of symptoms and diagnostic investigations were interviewed. Studies focusing on a homogeneous patient group could provide a more specific picture. Some issues emerging from the interviews in this study may be particularly relevant in healthcare systems, such as the British and similar systems, characterised by a strong GP gatekeeper role and rigid consultation norms.^39^ However, most factors highlighted in the present study can have a more general relevance for people receiving an all-clear, as also previously suggested.^21^,^40^ In this study, the term ‘delayed’ help seeking was used if participants waited for many weeks or months before seeking advice despite noticing symptoms. Different terms have been suggested, such as ‘postponement of help seeking’ or ‘prolonged intervals’.^41^ Each term has its own interpretation issues, however, and there is no consensus for defining appropriate patient intervals, especially in case of a previous all-clear diagnosis.

### Comparison with existing literature

The findings from the present study are in agreement with a recent systematic review.^21^ That review was limited, however, by the included studies not being designed for investigating the subject matter and mainly being based on reports of patients with cancer. Recalling information after receiving a cancer diagnosis could introduce a bias, as patients’ answers may be influenced by a sense of guilt or regret if they did not seek help promptly.^22^ A few studies included non-cancer patients.^42^–^45^,^23^ The information emerging from these studies on the impact of an all-clear diagnosis on help seeking was limited, but what emerged supports the present findings. For example, some females with post-menopausal bleeding after negative investigations reported a sense of frustration, not knowing what to do and feeling unable to seek help again for recurrent symptoms.^45^

The present findings are in line with existing theoretical models and psychological theory.^30^–^32^ In particular, in agreement with the model of pathways to treatment,^30^ the present study has highlighted the importance of considering the cyclical nature of the processes leading from symptom appraisal to help seeking to diagnosis, with the need to reappraise symptoms over time. The study emphasises how an all-clear diagnosis can play an important role influencing these ‘forward and backward movements’. Psychological theories of health behaviour, such as the SCT, can help to understand the specific contribution of a previous all-clear diagnosis on help seeking.^30^–^32^ In line with the SCT, the decision to seek help and the time before help seeking can be influenced by a patient’s perceived ability to discuss the symptom and receive help (‘self-efficacy’). Previous experience (mastery), social models, and social persuasion affect self-efficacy. No intention to help seeking will be developed if a patient has the perception that help is not available or the barriers are too difficult to overcome. This is in agreement with the present findings showing that some participants experiencing recurrent or new possible cancer symptoms after an all-clear diagnosis delayed help seeking as they were left with a sense of resignation and the perception that the doctor would not be able to help them. Many interviewees felt under-supported, because of an overburdened healthcare system, with difficulties in accessing primary and specialist care; lack of time during the consultation and the GP’s gatekeeper role were seen as relevant barriers preventing them from seeking help again after an all-clear.

On the other hand, SCT recognises that self-efficacy can be enhanced by opportunities, such as systems facilitating access to health care. The present study has shown that participants who had received specific advice, for example, on symptom monitoring, or had experienced uncomplicated access to health care and an empathetic doctor–patient relationship, felt motivated and supported to seek help promptly.

According to SCT, help seeking is also strongly influenced by ‘outcome expectations’: the perceived consequences of help seeking, in terms of physical, social, and self-evaluative outcomes, can act both as incentives and disincentives. For example, if a patient believes that seeing a doctor will improve their prognosis or will be useful for controlling a symptom or reducing anxiety, help seeking will be incentivised.^30^ On the other hand, people may be disincentivised if they believe that seeing a doctor may lead to unwanted investigations/treatments or to a sense of embarrassment because a symptom turned out to be a false alarm and they could be seen as neurotic or a time waster.^19^,^30^ The present study found that an all-clear diagnosis left many participants with the perception that if they returned to the doctor they could be seen as hypochondriacal or somebody who makes a fuss, which were important reasons preventing them from seeking help despite experiencing alarm symptoms. Fear of investigations and embarrassment because of symptoms in intimate areas were rarely mentioned as an explanation for delayed help seeking in the present study. A previous survey on patients with cancer also showed that embarrassment was not reported often, and was not among the most important reasons for explaining delayed presentation for cancer symptoms.^46^ Although embarrassment is often reported as a barrier for screening participation,^47^ it may be less relevant in preventing people from seeking help when experiencing symptoms; other factors, including reassurance from previous investigations, may be more important among symptomatic patients. It should be noted that, even though studies referring to screened and to symptomatic individuals can complement each other, the findings are not directly transferable.

### Implications for research and practice

Considering the significant increase in diagnostic investigations for potential cancer symptoms, and the concomitant rise in all-clear diagnoses observed over recent years,^10^ it is paramount to limit unintended consequences. This study has shown that a previous all-clear diagnosis can influence subsequent help seeking. This is an under-researched area and, even if the effect is small, it could impact on a large number of people. Further large prospective studies are needed to estimate the strength of the association and the impact at population level. Other factors can also influence help seeking, including symptom characteristics, comorbidities, social support, fatalism, or a patient’s ‘general attitude’ towards help seeking.^3^,^46^ More studies are needed to further examine these and other factors and their interaction. The present study, focusing specifically on a previous all-clear diagnosis, identified factors that could be addressed by healthcare providers to limit unintended consequences.

In particular, specific advice during/after health investigations are necessary to avoid leaving patients with a false sense of security or a sense of under-support. Despite the need and desire to reassure patients after negative investigations, it should be remembered that the diagnostic process is often iterative rather than linear^30^ and may require re-evaluating symptoms over time. Appropriate approaches for communicating with patients after an all-clear should be developed, finding a balance between the need to reassure patients and, at the same time, highlighting the importance of attending to bodily sensations. As part of a safety-netting approach, some general recommendations have been published.^48^,^49^ However, more specific advice and support is needed after an all-clear particularly for potentially recurrent or long-lasting problems. A study on lung cancer diagnosis^50^ reported that patients receiving explicit advice on symptom monitoring or on when to reconsult felt legitimised to seek help for persistent symptoms; however, patients did not commonly receive sufficient advice. Symptom monitoring has been highlighted as an important approach for expediting cancer diagnosis^51^ and it could be particularly useful in the case of recurrent symptoms after an all-clear diagnosis. Recommendations for safety-netting and preventing delays in cancer diagnosis^48^,^49^ include informing the patient that there is uncertainty and that more visits may be needed to reach a diagnosis, explaining what symptoms deserve special attention, how to seek help if necessary, and describing the expected development of the illness over time. Awareness should be raised about the importance of a doctor, rather than the patient alone, (re-)evaluating persistent, recurrent, or new symptoms, emphasising that this will not be a waste of the doctor’s or the patient’s time. Verbal and non-verbal doctor–patient communication, paying attention not to be dismissive and instead validating patients for having sought help even when symptoms turn out to be benign, can be important. The dual role of the GP as a patient advocate and as a gatekeeper can create tension,^52^ and can influence patient help seeking behaviour. An ecological study has shown that healthcare systems with a strong GP gatekeeper role have poorer cancer survival compared with other systems.^39^ More research is needed exploring these issues to identify approaches that can mitigate the negative effects and at the same time sustain the advantages of such systems.

A clinical encounter after negative diagnostic investigations may be useful to ensure that bodily sensations are not dismissed following negative examinations, and to discuss next steps in the case of recurrent or new symptoms. Moreover, planned follow-up can allow the clinician to re-evaluate possible alternative diagnoses, with symptom changes guiding this process.^53^ Negative investigations can sometimes also falsely reassure doctors and lead to referral delays.^54^,^55^ More evidence is needed for guiding doctors on the time intervals when further visits and investigations after an all-clear are warranted. It is worth emphasising that the risk of cancer after an all-clear diagnosis is not insignificant:^28^ according to previous research, 13% of breast cancers were diagnosed in females with a palpable mass within 1 year of a negative mammogram;^27^ audits reported that 19.5% of patients with lung cancer had an initial chest X-ray that did not show suspicion of cancer.^29^

The present study has identified specific issues that could be addressed to limit possible unintended consequences after an all-clear diagnosis. This is particularly important considering the significant increase of diagnostic investigations with an all-clear diagnosis in recent years. Appropriate information and support tools for patients and physicians could be developed to limit delays in case of new or recurrent cancer symptoms after an all-clear and to help diagnose cancer promptly.

## Appendix 1. Interview guide and list of main topics

**Table:**

**Main topic**	**Example question**
Review of type/number of symptoms	• Could you describe the symptoms you recently experienced? *Choose relevant symptoms from survey and continue questions.*
Exploration of key symptom attributes	• What characteristics of the symptom do you consider important? *Prompt around frequency/duration and severity, concern, etc.*
Symptom attribution	• What sorts of ideas go through your mind when you think about what caused the symptom?
Social context	• Do you know other people who have had similar symptoms?
Stoicism	• Do you think of yourself as someone who just ‘gets on with life’ despite your symptoms?
Serious symptoms	• What is it about the symptom that made you think it was serious?
Emotional response	• How did you feel about the symptom?
Disclosure/help seeking	• Did you talk to anyone about the symptom? What would prompt you to talk to a doctor about your symptom? *• If participant reported waiting before seeing a GP for a symptom*: What made you wait before seeing the GP/health professional for this symptom?
Family history/perceived risk of serious illness	• Do you have a family history of any serious illness?

## Appendix 2. Supplementary details on factors influencing delayed help seeking in relation to a previous all-clear diagnosis

**Table:**

**Theme**	**Subtheme**	**Examples**	**Patient characteristics**
Over-reassurance	Symptom normalised or attributed to previous benign diagnosis	*‘Especially since I had the scan, I’ve never really thought it’s going to be anything serious otherwise they would have spotted it … You see, the more investigations they did, it made me feel a bit easier … I’ve probably chosen to live with it …’*	M, 62 years; recurrent abdominal pain and bloating, for which delayed help seeking for the last 12 months after a previous colonoscopy with a benign diagnosis
	Perception of understanding the problem based on previous experience	*‘I just think I know what it is, so I don’t need to see a doctor’*	F, 53 years; rectal bleeding and previous diagnosis of haemorrhoids; despite worsening symptoms has not seen the doctor for 3 years
	Trust in diagnostic tests overruled bodily sensations	*‘They did some thorough tests on me, put the camera up … They didn’t think it was too serious because they did all these tests, you see … It reassured me that it wasn’t so bad, yeah …’*	M, 62 years; persistent and worsening bladder problem; has not mentioned problem to GP for past 4 years
Under-support	Worried about appearing hypochondriacal	*‘If you’ve already been told one solution, even if you don’t think it’s right, it does put you off going again … Well, not wanting to be a hypochondriac, to cause a fuss …’*	F, 72 years; recurrent chest pain and blood in sputum; waited more than 1 year before seeing doctor again after all-clear; has chronic emphysema
	Not wanting to waste doctor’s time/bother the doctor	*‘I wouldn’t want to bother the doctor again, and I would think, “Well, I’ve had a chest X-ray and they said it’s absolutely fine. This is just something that I’ve got to cope with.” And unless the symptoms became worse … then, no, I wouldn’t go back’*	F, 64 years; recurrent respiratory symptoms for more than 20 years and persistent cough; diagnosed with COPD; symptoms worsened over the last 2 years but has not sought help
	Humiliation, not being taken seriously	*‘ ... they just don’t have time to sit there and listen to what you want to say. I mean, sometimes I might go in with a piece of paper and write down my symptoms, for the doctor to push it away and not even look at it ... I thought they just don’t care … I don’t feel like going back when they are going to do that’*	F, 62 years; recurrent pain, history of breast lump with all-clear after mammogram; delayed help seeking
	Frustration, resignation, doctor unable to help	*‘If you’ve been to see the doctor and, as in my case, she says there’s nothing else she can do for me regarding that, it’s a bit pointless going and bothering her with something which she can’t do anything about, I suppose’*	M, 62 years; recurrent abdominal pain and bloating; delayed help seeking after a previous scan with benign diagnosis
	Perception of GP as gatekeeper	*‘I know that if I suggested, “Well, maybe do you think a scan?’, he’s just not going to put me forward because I don’t have any symptoms that would make it worthwhile … And so I feel like I wouldn’t get anywhere; I wouldn’t be taken that seriously, really …’*	M, 55 years; persistent gastrointestinal symptoms; delayed returning to GP for more than 2 years after an all-clear diagnosis
	Lack of communication/information	*‘… if I went through that process again I definitely would want the results, in that situation, I’d want to do it face-to-face … I’d then have the ability to ask questions on that. Because, to this day, I don’t know exactly what it is’*	M, 51 years; all-clear diagnosis after investigations for a lump; recurrent rectal bleeding; delayed help seeking for 6 years after previous diagnosis of haemorrhoids
	Anxiety, fear, embarrassment	*‘The only one where I would wait is that stomach one, because I don’t like that tube going down my throat … I wouldn’t bother about the one about my prostate … It all depends on the test. If it was a colonoscopy, I wouldn’t bother …’*	M, 67 years; recurrent rectal bleeding and urinary symptoms; no delays for recurrent symptoms after colonoscopy, but delays for upper GI symptoms

## References

[1] Berrino F, De Angelis R, Sant M (2007). Survival for eight major cancers and all cancers combined for European adults diagnosed in 1995–99: results of the EUROCARE-4 study. Lancet Oncol.

[2] Coleman MP, Forman D, Bryant H (2011). Cancer survival in Australia, Canada, Denmark, Norway, Sweden, and the UK, 1995–2007 (the International Cancer Benchmarking Partnership): an analysis of population-based cancer registry data. Lancet.

[3] Macleod U, Mitchell ED, Burgess C (2009). Risk factors for delayed presentation and referral of symptomatic cancer: evidence for common cancers. Br J Cancer.

[4] Mitchell E, Macdonald S, Campbell NC (2008). Influences on pre-hospital delay in the diagnosis of colorectal cancer: a systematic review. Br J Cancer.

[5] Department of Health (2012). Direct access to diagnostic tests for cancer: best practice referral pathways for general practitioners.

[6] Cancer Research UK Be clear on cancer.

[7] Canadian Cancer Society Finding cancer early.

[8] American Cancer Society Women’s health.

[9] World Health Organization (2007). Cancer control: knowledge into action — WHO guide for effective programmes Early detection.

[10] Public Health England National Cancer Intelligence Network Data Briefing. National trends in CWT metrics, 2009/10 to 2013/14.

[11] Montgomery M, McCrone SH (2010). Psychological distress associated with the diagnostic phase for suspected breast cancer: systematic review. J Adv Nurs.

[12] Andrykowski MA, Carpenter JS, Studts JL (2002). Psychological impact of benign breast biopsy: a longitudinal, comparative study. Health Psychol.

[13] Pelletier M, Knauper B, Loiselle CG (2012). Moderators of psychological recovery from benign cancer screening results. Curr Oncol.

[14] Witek-Janusek L, Gabram S, Mathews HL (2007). Psychologic stress, reduced NK cell activity, and cytokine dysregulation in women experiencing diagnostic breast biopsy. Psychoneuroendocrinology.

[15] Arndt V, Stürmer T, Stegmaier C (2003). Provider delay among patients with breast cancer in Germany: a population-based study. J Clin Oncol.

[16] Arndt V, Sturmer T, Stegmaier C (2002). Patient delay and stage of diagnosis among breast cancer patients in Germany — a population based study. Br J Cancer.

[17] Hogberg C, Karling P, Rutegard J (2013). Immunochemical faecal occult blood tests in primary care and the risk of delay in the diagnosis of colorectal cancer. Scand J Prim Health Care.

[18] Macdonald S, Macleod U, Campbell NC (2006). Systematic review of factors influencing patient and practitioner delay in diagnosis of upper gastrointestinal cancer. Br J Cancer.

[19] Smith LK, Pope C, Botha JL (2005). Patients’ help seeking experiences and delay in cancer presentation: a qualitative synthesis. Lancet.

[20] Heisey R, Clemons M, Granek L (2011). Health care strategies to promote earlier presentation of symptomatic breast cancer: perspectives of women and family physicians. Curr Oncol.

[21] Renzi C, Whitaker KL, Wardle J (2015). Over-reassurance and undersupport after a ‘false alarm’: a systematic review of the impact on subsequent cancer symptom attribution and help seeking. BMJ Open.

[22] Solbjor M, Skolbekken JA, Saetnan AR (2012). Could screening participation bias symptom interpretation? An interview study on women’s interpretations of and responses to cancer symptoms between mammography screening rounds. BMJ Open.

[23] Janz NK, Becker MH, Haefner DP (1990). Determinants of breast self-examination after a benign biopsy. AmJ Prev Med.

[24] Vaughan-Shaw PG, Cutting JE, Borley NR, Wheeler JM (2013). Repeat 2-week wait referrals for colorectal cancer. Colorectal Dis.

[25] Shin S, Schneider HB, Cole FJ, Laronga C (2006). Follow-up recommendations for benign breast biopsies. Breast J.

[26] Goldacre MJ, Abisgold JD, Yeates DG, Vessey MP (2010). Benign breast disease and subsequent breast cancer: English record linkage studies. J Public Health.

[27] Haakinson DJ, Stucky CC, Dueck AC (2010). A significant number of women present with palpable breast cancer even with a normal mammogram within 1 year. Am J Sur.

[28] Dominitz JA, Robertson DJ (2013). Editorial: interval cancers: learning from the past as we build for the future. Am J Gastroenterol.

[29] Neal RD, Robbe IJ, Lewis M (2015). The complexity and difficulty of diagnosing lung cancer: findings from a national primary-care study in Wales. Prim Health Care Res Dev.

[30] Scott SE, Walter FM, Webster A (2013). The model of pathways to treatment: conceptualization and integration with existing theory. Br J Health Psychol.

[31] Bandura A (1998). Health promotion from the perspective of social cognitive theory. Psychol Health.

[32] Bandura AS (1997). Self-efficacy: the exercise of control.

[33] Whitaker KL, Cromme S, Winstanley K (2015). Emotional responses to the experience of cancer ‘alarm’ symptoms. Psychooncology.

[34] Whitaker KL, Macleod U, Winstanley K (2015). Help seeking for cancer ‘alarm’ symptoms: a qualitative interview study of primary care patients in the UK. Br J Gen Pract.

[35] Whitaker KL, Scott SE, Winstanley K (2014). Attributions of cancer ‘alarm’ symptoms in a community sample. PloS One.

[36] Teuschl Y, Brainin M (2010). Stroke education: discrepancies among factors influencing prehospital delay and stroke knowledge. Int J Stroke.

[37] Brindle L, Pope C, Corner J (2012). Eliciting symptoms interpreted as normal by patients with early-stage lung cancer: could GP elicitation of normalised symptoms reduce delay in diagnosis? Cross-sectional interview study. BMJ Open.

[38] Green J, Thorogood N (2009). Qualitative methods for health research.

[39] Vedsted P, Olesen F (2011). Are the serious problems in cancer survival partly rooted in gatekeeper principles? An ecologic study. Br J Gen Pract.

[40] Lyratzopoulos G, Vedsted P, Singh H (2015). Understanding missed opportunities for more timely diagnosis of cancer in symptomatic patients after presentation. Br J Cancer.

[41] Dobson CM, Russell AJ, Rubin GP (2014). Patient delay in cancer diagnosis: what do we really mean and can we be more specific?. BMC Health Serv Res.

[42] Beacham AO, Carpenter JS, Andrykowski MA (2004). Impact of benign breast biopsy upon breast self-examination. Prev Med.

[43] Facione NC, Facione PA (2006). The cognitive structuring of patient delay in breast cancer. Soc Sci Med.

[44] Jones SC, Gregory P, Nehill C (2010). Australian women’s awareness of breast cancer symptoms and responses to potential symptoms. Cancer Causes Control.

[45] Tarling R, Gale A, Martin-Hirsch P (2013). Experiences of women referred for urgent assessment of postmenopausal bleeding (PMB). J Obstet Gynaecol.

[46] Forbes LJ, Warburton F, Richards MA, Ramirez AJ (2014). Risk factors for delay in symptomatic presentation: a survey of cancer patients. Br J Cancer.

[47] Waller J, Bartoszek M, Marlow L, Wardle J (2009). Barriers to cervical cancer screening attendance in England: a population-based survey. J Med Screen.

[48] Mitchell ED, Rubin G, Macleod U (2013). Understanding diagnosis of lung cancer in primary care: qualitative synthesis of significant event audit reports. Br J Gen Pract.

[49] Almond S, Mant D, Thompson M (2009). Diagnostic safety-netting. Br J Gen Pract.

[50] Birt L, Hall N, Emery J (2014). Responding to symptoms suggestive of lung cancer: a qualitative interview study. BMJ Open Respir Res.

[51] Walter FM, Rubin G, Bankhead C (2015). Symptoms and other factors associated with time to diagnosis and stage of lung cancer: a prospective cohort study. Br J Cancer.

[52] Jiwa M, Reid J, Handley C (2004). Less haste more speed: factors that prolong the interval from presentation to diagnosis in some cancers. Fam Pract.

[53] Berner ES, Graber ML (2008). Overconfidence as a cause of diagnostic error in medicine. Am J Med.

[54] Mitchell ED, Rubin G, Merriman L, Macleod U (2015). The role of primary care in cancer diagnosis via emergency presentation: qualitative synthesis of significant event reports. Br J Cancer.

[55] Evans J, Chapple A, Salisbury H (2014). ‘It can’t be very important because it comes and goes’ — patients’ accounts of intermittent symptoms preceding a pancreatic cancer diagnosis: a qualitative study. BMJ Open.

